# Reply to ‘Interpreting the clinical utility and generalizability of a multitask perioperative prediction model’

**DOI:** 10.1038/s41746-026-02995-7

**Published:** 2026-08-03

**Authors:** Hyun-Kyu Yoon, Hyung-Chul Lee, Hyeonhoon Lee

**Affiliations:** 1 Department of Anesthesiology and Pain Medicine, Seoul National University Hospital, Seoul, Republic of Korea; 2 Department of Anesthesiology and Pain Medicine, Seoul National University College of Medicine, Seoul, Republic of Korea; 3 Healthcare AI Research Institute, Seoul National University Hospital, Seoul, Republic of Korea; 4 Department of Transdisciplinary Medicine, Seoul National University Hospital, Seoul, Republic of Korea; 5 Department of Medicine, Seoul National University College of Medicine, Seoul, Republic of Korea

**Keywords:** Biomarkers, Computational biology and bioinformatics, Diseases, Medical research, Nephrology, Risk factors

## Abstract

We thank Wo and Zhang for their comments on our multitask prediction model for postoperative complications, including postoperative acute kidney injury, respiratory failure, and in-hospital mortality. Here, we responded by mapping specific threshold probabilities to specific clinical actions in the decision curve analyses, quantifying the net benefit to express clinical gains as the number of additional true events detected per 1000 patients, and providing detailed calibration metrics with recalibration guidance.

We appreciated the thoughtful comments from Wo and Zhang regarding the clinical utility and generalizability of our multitask gradient-boosting machine (MT-GBM) model. Their questions provide an opportunity to further clarify the practical implications of our findings for perioperative risk stratification.

## Response to Question 1

Wo and Zhang appropriately note that the clinical decisions underlying decision curve analyses should be explicitly defined. We agree that linking threshold probabilities to specific clinical actions enhances the interpretability of our results, and we appreciate the opportunity to clarify our analytical approach.

The threshold probability in decision curve analysis represents the point at which a clinician would consider the expected benefit of intervention to equal the expected benefit of avoiding the intervention^1,2^. While our original analysis plotted threshold probabilities from 0% to 50% to show overall model performance, we acknowledge that clinically actionable thresholds are concentrated in narrower, outcome-specific ranges where preventive interventions are both feasible and proportionate to the predicted risk.

For each outcome, we identified clinically relevant threshold ranges based on the intensity and resource requirements of the corresponding interventions (Table 1). For acute kidney injury (AKI), thresholds of 5–20% correspond to escalating nephroprotective strategies, ranging from enhanced renal monitoring at lower thresholds to comprehensive Kidney Disease: Improving Global Outcomes (KDIGO) bundle implementation with nephrology consultation at higher thresholds^3,4^. For postoperative respiratory failure (PRF), thresholds of 2–10% reflect increasing intensity of pulmonary care, from preoperative respiratory education at lower thresholds to comprehensive pulmonary care bundle assignment of an experienced provider, and planned intensive care admission at higher thresholds^5–8^. For in-hospital mortality, thresholds of 1–5% guide decisions from enhanced postoperative monitoring to goals-of-care discussion^9–11^.

**Table 1:** Risk threshold-based clinical decision framework

Outcome	Threshold	Clinical decision	Specific interventions	Derivation Cohort	Validation Cohort A	Validation Cohort B
Acute kidney injury	≥5%	Enhanced renal monitoring	Frequent serum creatinine measurements, urine output tracking	12.9%	15.0%	17.0%
	≥10%	Active nephroprotective measures	Nephrotoxic drug avoidance, hemodynamic optimization	6.6%	8.6%	8.8%
	≥20%	Comprehensive KDIGO bundle	Nephrology consultation, full KDIGO bundle implementation	2.6%	4.2%	3.7%
Postoperative respiratory failure	≥2%	Enhanced respiratory monitoring	Preoperative incentive spirometry education, smoking cessation counseling	5.0%	6.9%	8.8%
	≥5%	Pulmonary optimization	Preoperative pulmonary optimization, lung-protective ventilation	2.7%	4.2%	5.8%
	≥10%	Comprehensive pulmonary care bundle	Experienced provider assignment, planned ICU admission, multimodal prehabilitation	1.6%	2.7%	3.8%
In-hospital mortality	≥1%	Enhanced postoperative monitoring	Enhanced vital signs surveillance	5.9%	8.6%	11.9%
	≥2%	Intensivist consultation	Perioperative medical management optimization	3.8%	6.0%	8.7%
	≥5%	Goals-of-care discussion	Discussion of treatment preferences, expected outcomes, advance care planning	1.8%	3.5%	5.2%

Values represent the percentage of patients exceeding each risk threshold based on spline-calibrated multitask gradient-boosting machine model predictions

*KDIGO* Kidney Disease Improving Global Outco, *ICU* intensive care unit

We acknowledge that our original presentation did not explicitly link these threshold ranges to specific clinical decisions. We hope that this clarification helps readers interpret the decision curve analyses in the context of actual perioperative clinical practice. Future prospective studies examining whether model-guided decisions at these specific thresholds improve patient outcomes would further strengthen the evidence for clinical implementation.

## Response to Question 2

Wo and Zhang raise a valid point about interpreting model performance for rare outcomes. We agree that presenting absolute numbers alongside discrimination metrics better illustrates practical utility. In response, we calculated positive predictive values (PPVs), negative predictive values (NPVs), and the numbers of true positives and false positives per 1,000 patients at clinically relevant thresholds. We compared the MT-GBM model with American Society of Anesthesiologists (ASA) class ≥ 3 across these metrics (Table 2).

**Table 2:** Net benefit analysis comparing the multitask gradient-boosting machine model (MT-GBM) with the American Society of Anesthesiologists (ASA) physical status classification

Outcome	Threshold	Cohort	Model	PPV	NPV	TP/1000	FP/1000	NB/1000	ΔTP	ΔFP	ΔNB
**Acute kidney injury**	5%	Derivation	MT-GBM	11.7%	98.3%	15.1	113.7	9.15	+14.2	+107.9	+8.54
			ASA ≥ 3	13.6%	97.1%	0.9	5.8	0.61	(ref)	(ref)	(ref)
		Validation A	MT-GBM	13.7%	97.8%	20.5	129.4	13.66	−3.6	−80.4	+0.61
			ASA ≥ 3	10.3%	98.0%	24.1	209.8	13.05	(ref)	(ref)	(ref)
		Validation B	MT-GBM	14.0%	98.6%	23.8	145.8	16.15	+12.4	+121.6	+6.05
			ASA ≥ 3	32.0%	97.5%	11.4	24.2	10.10	(ref)	(ref)	(ref)
	10%	Derivation	MT-GBM	16.1%	97.9%	10.7	55.6	4.49	+9.8	+49.8	+4.22
			ASA ≥ 3	13.6%	97.1%	0.9	5.8	0.27	(ref)	(ref)	(ref)
		Validation A	MT-GBM	17.0%	97.3%	14.7	71.8	6.70	+8.7	+53.4	+2.72
			ASA ≥ 3	24.7%	96.6%	6.0	18.4	3.98	(ref)	(ref)	(ref)
		Validation B	MT-GBM	23.0%	98.4%	20.3	67.9	12.72	+8.9	+43.7	+4.03
			ASA ≥ 3	32.0%	97.5%	11.4	24.2	8.69	(ref)	(ref)	(ref)
	20%	Derivation	MT-GBM	23.8%	97.5%	6.1	19.7	1.21	+6.1	+19.7	+1.21
			ASA ≥ 3	0.0%	97.0%	0.0	0.0	0.00	(ref)	(ref)	(ref)
		Validation A	MT-GBM	17.5%	96.6%	7.3	34.3	−1.28	+1.3	+15.9	−2.71
			ASA ≥ 3	24.7%	96.6%	6.0	18.4	1.43	(ref)	(ref)	(ref)
		Validation B	MT-GBM	35.6%	97.7%	13.2	23.8	7.20	+1.8	−0.4	+1.87
			ASA ≥ 3	32.0%	97.5%	11.4	24.2	5.33	(ref)	(ref)	(ref)
**Postoperative respiratory failure**	2%	Derivation	MT-GBM	9.0%	99.5%	4.5	45.6	3.61	−1.5	−214.1	+2.93
			ASA ≥ 3	2.2%	99.5%	6.0	259.7	0.68	(ref)	(ref)	(ref)
		Validation A	MT-GBM	16.7%	99.4%	11.6	57.9	10.41	−2.7	−161.7	+0.59
			ASA ≥ 3	6.1%	99.6%	14.3	219.6	9.82	(ref)	(ref)	(ref)
		Validation B	MT-GBM	10.5%	99.5%	9.2	78.6	7.64	+0.7	+51.6	−0.34
			ASA ≥ 3	24.0%	99.4%	8.5	27.0	7.98	(ref)	(ref)	(ref)
	5%	Derivation	MT-GBM	13.2%	99.4%	3.6	23.5	2.32	+2.9	+17.5	+1.95
			ASA ≥ 3	10.2%	99.1%	0.7	6.0	0.37	(ref)	(ref)	(ref)
		Validation A	MT-GBM	23.7%	99.2%	9.9	32.0	8.25	+1.8	+15.7	+0.98
			ASA ≥ 3	33.3%	99.0%	8.1	16.3	7.27	(ref)	(ref)	(ref)
		Validation B	MT-GBM	14.8%	99.4%	8.5	49.1	5.95	0.0	+22.1	−1.16
			ASA ≥ 3	24.0%	99.4%	8.5	27.0	7.11	(ref)	(ref)	(ref)
	10%	Derivation	MT-GBM	17.3%	99.3%	2.7	13.0	1.28	+2.0	+7.0	+1.26
			ASA ≥ 3	10.2%	99.1%	0.7	6.0	0.02	(ref)	(ref)	(ref)
		Validation A	MT-GBM	30.1%	99.0%	8.2	19.0	6.09	+0.1	+2.7	−0.23
			ASA ≥ 3	33.3%	99.0%	8.1	16.3	6.32	(ref)	(ref)	(ref)
		Validation B	MT-GBM	19.4%	99.3%	7.5	30.9	4.03	−1.0	+3.9	−1.50
			ASA ≥ 3	24.0%	99.4%	8.5	27.0	5.53	(ref)	(ref)	(ref)
**In-hospital mortality**	1%	Derivation	MT-GBM	5.4%	99.8%	3.2	55.5	2.62	−0.1	−207.0	+2.02
			ASA ≥ 3	1.2%	99.7%	3.3	262.5	0.60	(ref)	(ref)	(ref)
		Validation A	MT-GBM	10.3%	99.4%	8.9	77.2	8.10	−2.7	−145.1	−1.25
			ASA ≥ 3	5.0%	99.7%	11.6	222.3	9.35	(ref)	(ref)	(ref)
		Validation B	MT-GBM	14.3%	98.7%	17.1	102.4	16.03	−7.1	−324.9	−3.83
			ASA ≥ 3	5.4%	99.2%	24.2	427.3	19.86	(ref)	(ref)	(ref)
	2%	Derivation	MT-GBM	7.2%	99.7%	2.7	35.2	2.01	+2.1	+29.1	+1.53
			ASA ≥ 3	9.1%	99.5%	0.6	6.1	0.48	(ref)	(ref)	(ref)
		Validation A	MT-GBM	12.6%	99.3%	7.5	52.2	6.46	−4.1	−170.1	−0.60
			ASA ≥ 3	5.0%	99.7%	11.6	222.3	7.06	(ref)	(ref)	(ref)
		Validation B	MT-GBM	17.2%	98.5%	14.9	71.8	13.47	−9.3	−355.5	−1.98
			ASA ≥ 3	5.4%	99.2%	24.2	427.3	15.45	(ref)	(ref)	(ref)
	5%	Derivation	MT-GBM	9.1%	99.6%	1.7	16.6	0.79	+1.1	+10.5	+0.50
			ASA ≥ 3	9.1%	99.5%	0.6	6.1	0.29	(ref)	(ref)	(ref)
		Validation A	MT-GBM	16.6%	99.1%	5.7	28.8	4.20	+0.4	+9.7	−0.06
			ASA ≥ 3	21.6%	99.1%	5.3	19.1	4.26	(ref)	(ref)	(ref)
		Validation B	MT-GBM	20.4%	98.1%	10.7	41.6	8.48	+3.9	+12.8	+3.24
			ASA ≥ 3	19.0%	97.7%	6.8	28.8	5.24	(ref)	(ref)	(ref)

*ASA* American Society of Anesthesiologists physical status classification, *PPV* positive predictive value, *NPV* negative predictive value, *TP* true positives, *FP* false positives, *NB* net benefit, *ΔTP* additional true positives vs. ASA class ≥3, *ΔFP* additional false positives vs. ASA class ≥3, *ΔNB* additional net benefit vs. ASA class ≥3. All values per 1000 patients unless otherwise specified

For AKI at the 5% threshold, the MT-GBM model showed net benefit improvements of +8.5, +0.6, and +6.1 per 1,000 patients across derivation, Validation A, and Validation B cohorts, respectively. In the derivation and Validation B cohorts, this benefit came from detecting more true positives (an additional 14.2 and 12.4 per 1000). In Validation Cohort A, it instead came from generating fewer false positives (80.4 fewer per 1,000). For PRF, the MT-GBM model showed consistent net benefit advantages at lower thresholds (2% and 5%) in the derivation and Validation A cohorts, with improvements ranging from +0.6 to +2.9 per 1,000 patients, primarily driven by reduced false-positive rates compared to ASA class ≥ 3. For in-hospital mortality, the MT-GBM model demonstrated a net benefit in the derivation cohort (+2.0 per 1000 at the 1% threshold, +1.5 at the 2% threshold). In external validation cohorts, however, the net benefit was comparable or slightly favored ASA class ≥ 3, suggesting that for this outcome, the advantage of the MT-GBM model may be less pronounced in populations with different case-mix characteristics.

These findings illustrate the complex trade-offs in risk prediction. While ASA class ≥ 3 captures more events at the cost of substantially more false alarms for outcomes like mortality, it under-predicts events for outcomes like AKI. For rare outcomes where the cost of unnecessary intervention is substantial, the MT-GBM model provides a clinically appropriate balance between event detection and false alarms, minimizing unnecessary clinical burden.

To aid interpretation, net benefit can be re-expressed as a count: a net-benefit advantage of, for example, 0.8 per 1000 patients corresponds to approximately 8 additional true positives identified per 10,000 surgeries at no increase in false positives. From a clinical perspective, for rare perioperative outcomes, such per-1000 differences in net benefit, though numerically small, can add up to clinically meaningful numbers at typical institutional surgical volumes. Furthermore, a count-based expression is more intuitive at the point of care than the net-benefit unit itself. The interpretability of a given net-benefit value nonetheless depends on the chosen risk threshold and the relative harms of false-positive and false-negative classifications it encodes.

We further note that qualitative evaluation of how clinicians interpret these net-benefit estimates in practice will be a valuable step prior to prospective clinical implementation.

## Response to Question 3

Wo and Zhang inquire about calibration performance in external cohorts and recommendations for local implementation. Regarding calibration performance after spline recalibration, calibration intercepts remained near zero across all outcomes (0.003–0.023), indicating minimal systematic bias in mean predicted probabilities. Calibration slopes were near-optimal for in-hospital mortality prediction in external validation cohorts (0.978–0.994), while AKI and PRF showed moderate overdispersion with slopes of 0.477–0.888. Calibration slopes below 1.0 for AKI and PRF indicate overdispersion, with the model overestimating risk in higher-risk patients and underestimating risk in lower-risk patients. However, this pattern is more pronounced at predicted probabilities exceeding 30%, which is beyond the clinically actionable threshold ranges described in our response to Question 1. Because the impact of miscalibration on decision-making is itself threshold-dependent, miscalibration confined to this high-risk range has little effect on the targeted clinical decisions^12,13^. Moreover, within this subset, the residual error is overestimation rather than underestimation, which is the safer direction in a perioperative setting because it favors closer monitoring or escalation of an already high-risk patient. This is consistent with the risk threshold encoding the relative harms of false-positive and false-negative classifications, with undetected cases generally considered more harmful than false-positive cases for severe outcomes^13,14^, and with failure-to-rescue evidence that timely identification and escalation of high-risk surgical patients reduce mortality^10^. Despite variable slopes, low Brier scores of 0.005–0.038 support the model’s overall predictive accuracy.

For institutions implementing MT-GBM, we recommend tailoring the approach based on local patient population characteristics using the two key diagnostics, including calibration-in-the-large (summarized by the calibration intercept, with a target value of 0, or the observed/expected ratio) and the calibration slope (target value 1, with a slope below 1 indicating risks that are too extreme)^14–16^. When both are close to the target, the model can be applied as published, indicating that the local case-mix and baseline risk resemble our derivation cohort. When only calibration-in-the-large is off, because the populations differ mainly in baseline event rate, intercept adjustment (the simplest update, re-estimating only the intercept) is sufficient and requires little local data. Conversely, if the calibration slope departs from 1, intercept-and-slope adjustment (logistic recalibration), in which the intercept is re-estimated and the predictor coefficients are multiplied by the calibration slope, is preferable. For populations where predictor effects or case-mix differ substantially, full re-estimation of all coefficients may be warranted, although this effectively develops a new model and requires a sufficiently large sample to avoid overfitting. Calibration should be reassessed after updates and monitored over time because local populations drift.

## Conclusion

We appreciate the opportunity to clarify these points, which support the foundation for prospective evaluation of model-guided perioperative risk management.

## Data Availability

The dataset used in this study is not publicly available. However, the data of this study can be provided if there is a reasonable request to the corresponding author.
